# Recurrent Spontaneous Pneumothorax Secondary to Marijuana and Tobacco Abuse

**DOI:** 10.7759/cureus.52391

**Published:** 2024-01-16

**Authors:** Naisargee N Solanki, Charlotte A Thill, Mahmoud Chaker, Angelo A Messina Alvarez, Nouraldeen Manasrah, Ahmed Jamal Chaudhary

**Affiliations:** 1 Internal Medicine, Detroit Medical Center/Wayne State University (DMC/WSU) Sinai Grace Hospital, Detroit, USA; 2 Internal Medicine, School of Medicine, Wayne State University, Detroit, USA

**Keywords:** smoking and postoperative outcomes, video-assisted thoracoscopic surgery (vats), recurrent pneumothorax, marijuana and pneumothorax, smoking and pneumothorax, tobacco and marijuana use, tension pneumothorax, marijuana abuse, primary spontaneous pneumothorax

## Abstract

Primary spontaneous pneumothorax occurs in patients without apparent clinical lung disease, with a higher incidence in tall, thin males between the ages of 10 and 30. Tension pneumothorax is a life-threatening condition that can develop within minutes due to progressive air accumulation in the pleural space; mechanical pressure can lead to significant cardiorespiratory compromise. Tobacco association with a higher incidence of spontaneous pneumothorax has been well documented, but marijuana and spontaneous pneumothorax connection has not been well studied. However, it has been observed that patients who use marijuana and tobacco simultaneously have a higher incidence of spontaneous tension and larger pneumothoraces, as well as longer postoperative stay and higher recurrence than cigarette-only users. We present a case of a 26-year-old young male with a history only significant for excessive tobacco and marijuana smoking who developed multiple recurrent spontaneous pneumothorax and had to undergo right-sided video-assisted thoracoscopic surgery (VATS) with minimally invasive thoracotomy and had a prolonged hospital stay. With our case report, we hope to add to the evidence the effects of combined marijuana and tobacco smoking on bullous lung disease and pneumothorax while emphasizing the importance of conducting a detailed substance use history in patients with spontaneous pneumothorax.

## Introduction

Marijuana is the second most commonly smoked substance in our society after tobacco, and its use has been increasing over the years. One study using the U.S. National Surveys on Drug Use and Health (NSDUH) data found that the prevalence of past-year cannabis use among adults in the United States went from 10.4% (or 21.9 million) in 2002 to 15.3% (or 37.8 million) in 2017, a 72.6% rise in the number of adult cannabis users in the country [1]. Additionally, in the states that are legalizing the recreational use of marijuana, rates of marijuana use have continued to rise after legalization [2]. This increase in use could be because of the availability of more potent cannabis products, which are more readily available at a lower price in states that have legalized the recreational use of marijuana [3]. Although the deleterious effects of tobacco smoke are well known, the effects of marijuana use are not well known. Marijuana smoking has been linked to chronic bronchitis, but its association with other lung diseases is not well established [4]. One such disease is pneumothorax. A primary spontaneous pneumothorax occurs in patients without apparent clinical lung disease, with a higher incidence in tall, thin boys and men between the ages of 10 and 30 years [5]. Tension pneumothorax is a life-threatening condition that can develop within minutes. Due to progressive air accumulation in the pleural space, mechanical pressure can lead to significant respiratory compromise and cardiovascular collapse [6]. The effects of tobacco smoking on spontaneous pneumothorax are well described; however, the effects of marijuana-only smoking or combined smoking of tobacco and marijuana have not been well defined [7]. We report a case of a 26 year-old-male with a significant history of marijuana and tobacco smoking diagnosed with tension pneumothorax and recurrent spontaneous pneumothorax. With this case report, we hope to add to the evidence the effects of combined marijuana and tobacco smoking on bullous lung disease and pneumothorax while emphasizing the importance of conducting a detailed substance use history in patients with spontaneous pneumothorax. This article was previously presented as a poster presentation at the 2022 ACP annual scientific meeting - Michigan chapter on October 15, 2022.

## Case presentation

A 26-year-old male with a history of using multiple substances presented to the emergency department complaining of difficulty breathing and pain on the right side of his chest. Three days prior, the patient had developed a small pneumothorax and was treated with a pigtail catheter placement at the hospital and subsequently discharged. On the same day as his discharge, he developed similar symptoms after coughing and sneezing. As his breathing worsened and chest pain increased, he decided to seek care in our emergency department. The patient had no significant medical history except for substance abuse, smoking seven to eight cigars, and smoking 7 g of marijuana daily for 10 to 12 years. 

**Figure 1:**
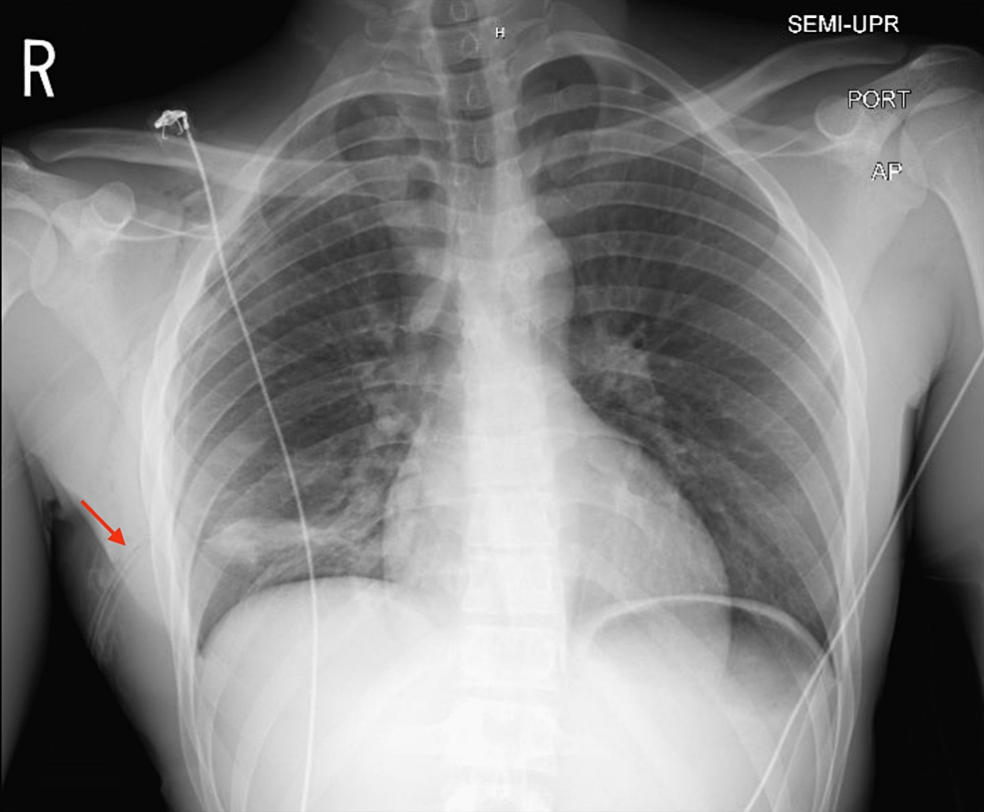
Chest x-ray after right chest tube placement

**Figure 2:**
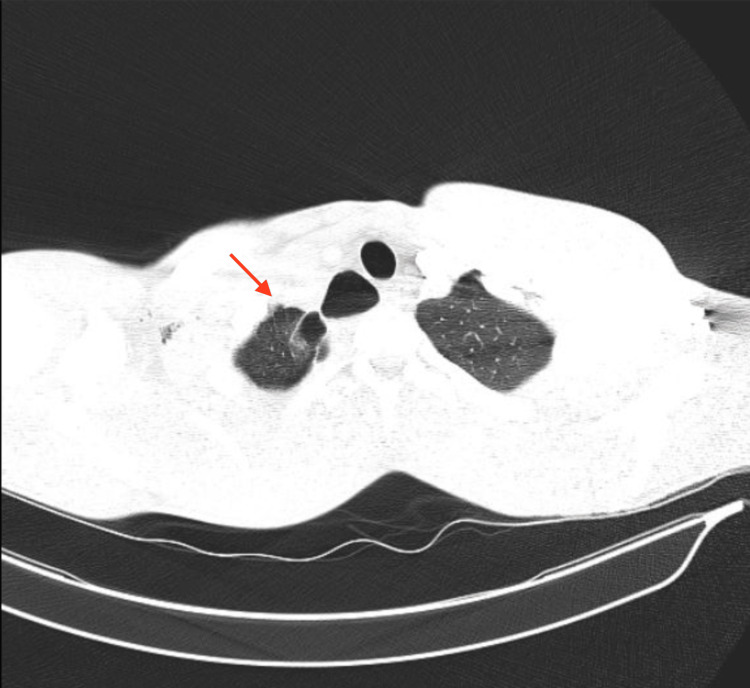
CT thorax with contrast: multiple right apical blebs and residual right pneumothorax

Initial assessment indicated breathing difficulty, rapid breathing at a rate of 32 breaths per minute, high blood pressure at 161/110 mm Hg, full oxygen saturation while using a nonrebreather mask with 15 L of oxygen, heart rate of 68 beats per minute, swollen neck veins on the right side, and shallow breathing with no sounds from the right lung. Physical examination pertaining to marfanoid features was unremarkable, with a height of 187.96 cm, weight of 71 kg, and body mass index (BMI) of 20.1 kg/m^2^. A point-of-care ultrasound revealed a lack of movement in the right lung during breathing. The absence of sound from the right lung and the presence of swollen neck veins on that side raised concerns about the development from acute spontaneous pneumothorax to tension pneumothorax, which required immediate insertion of a chest tube. Upon tube placement, there was a significant and prolonged rush of air with moderate relief of symptoms and continued chest pain. After the procedure, the patient experienced increased right-sided pain, so a new chest tube was placed more anteriorly under sedation. Laboratory findings were significant for leukocytosis of 12.5 × 10^9^/L and high alpha 1 antitrypsin level of 189 mg/dL (reference range 95-164 mg/dL). After chest tube placement, the chest x-ray showed a stable chest with a small right apical pneumothorax and lower right lung atelectasis (Figure 1). Further investigation with a CT thorax with contrast showed multiple right apical blebs, a right thoracostomy tube, and residual right pneumothorax (Figure 2).

Based on the CT thorax results, the cardiothoracic surgery team scheduled a minimally invasive right-sided video-assisted thoracoscopic surgery (VATS) procedure with thoracotomy to address recurrent spontaneous pneumothorax and right apical blebs. Resection of a large bleb on the posterior-lateral right upper lobe and multiple blebs along the medial right upper lobe was performed. Pathology confirmed pleural blebs associated with acute fibrino-purulent pleuritis and organizing pneumonia of the subpleural lung. While attempting to free the adhesion between the right apical lung and pleura using blunt dissection, it failed; therefore, mini-thoracotomy over the fifth intercostal space had to be done for this purpose as well as freeing apical blebs. Mechanical pleurodesis of the circumferential area around the right lung followed it.

**Figure 3:**
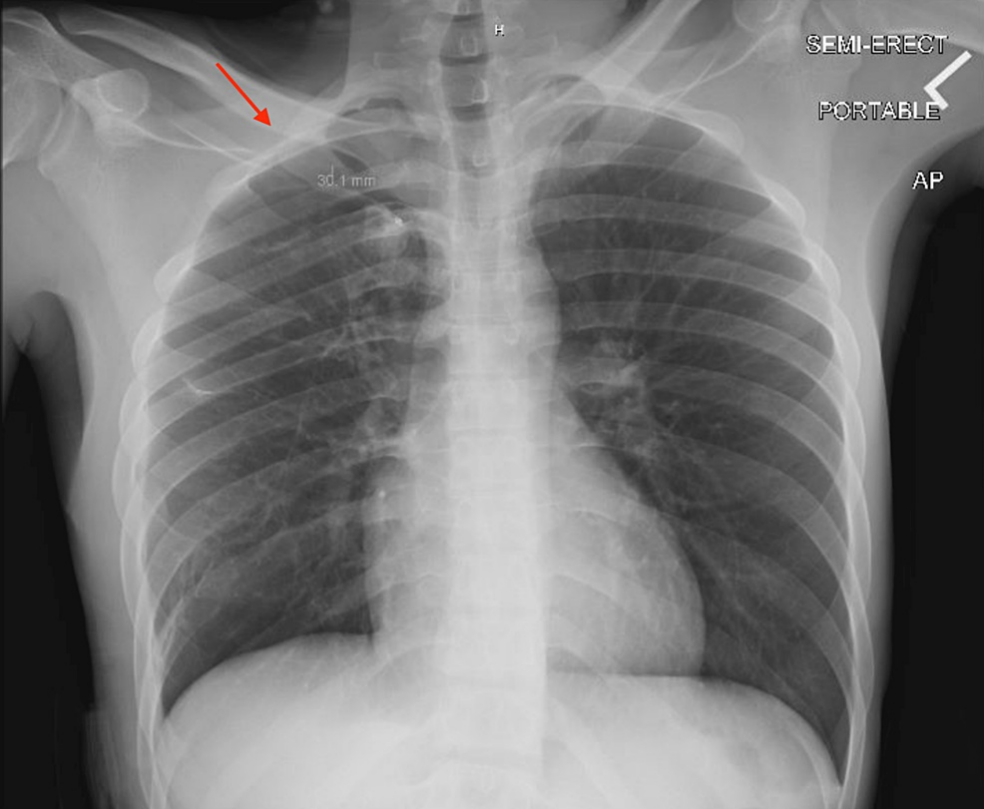
Chest X-ray showing stable small right apical pneumothorax

Following the surgery, imaging revealed that the patient had a minor residual pneumothorax, which was treated with chest tubes connected to a water seal. The patient was transferred to the surgical intensive care unit and treated with pain control and incentive spirometry use. Prior to discharge, the right chest tube was removed, and a subsequent chest x-ray showed a stable small right apical pneumothorax (Figure 3). The patient received counseling on the negative effects of marijuana and tobacco use, along with encouragement to cease both habits and was discharged home after 15 days postoperation and a total hospital stay of 23 days.

## Discussion

The association between tobacco and a higher incidence of spontaneous pneumothorax has been well documented, but the connection between marijuana and spontaneous pneumothorax has not been clearly defined [8]. Several case reports support the link between pneumothorax and marijuana smoking [9,10,11]. Marijuana smoking is linked to airflow obstruction, impaired large airway function, and hyperinflation in a dose-dependent manner. One joint of marijuana has a similar effect to 2.5-5 tobacco cigarettes in terms of causing airflow obstruction [12]. Marijuana smokers typically use deep inhalation techniques with prolonged breath-holding time. They have a 66% larger puff volume, a 33% increase in breathing depth and inhalation time, as well as four times longer breath-holding [8,13]. Some may perform Valsalva or muller maneuvers during their breath-holding technique, which could potentially contribute to alveolar rupture and air leak [14,15]. Additionally, marijuana is usually smoked without filters and to shorter butt lengths [16,17]. All these factors could contribute to worsening outcomes in patients who are combined smokers. 

A case-control study in Denmark found that the risk of primary spontaneous pneumothorax significantly increases in young men who combine smoking tobacco and cannabis compared to those who only smoke tobacco [18]. Another case-control study by Hedevang Olesen et al. found that male patients who combined daily cannabis and tobacco smoking had a higher chance of experiencing a primary spontaneous pneumothorax compared to those who only smoked tobacco, with a five-fold increase for smokers vs. never smokers and a nine-fold increase for combined smoking vs. never smokers [7,18]. Additionally, another study showed that patients with pneumothorax who are both tobacco and cannabis smokers develop emphysema at an earlier age than those who only smoke tobacco [19]. A retrospective case-control study on patients operated on for spontaneous pneumothorax found that marijuana smoking has detrimental effects on lung parenchyma in a dose-dependent manner. They have a higher risk of developing larger pneumothorax and longer postoperative stays compared to non-smokers [8]. 95% of subjects in the study who smoked marijuana also smoked tobacco. Compared to tobacco-only smokers, marijuana smokers had a two times higher incidence of recurrence and 0.7 days longer postoperative stay. However, these findings were not statistically significant, most likely due to the smaller sample size of 112 subjects. 

Our patient, a 26-year-old male, had no significant medical/surgical/family history except for heavy marijuana and tobacco use. The physical exam did not indicate marfanoid features and the antitrypsin levels were elevated. CT thorax showed multiple blebs in the right lung and no other structural abnormalities, leading to the diagnosis of primary recurrent pneumothorax. Heavy marijuana and nicotine consumption were identified as potential contributors to bullous lung disease development resulting in his pneumothorax. Following this diagnosis, the patient underwent right-sided VATS and a mini-thoracotomy to free the right lung completely due to recurrent spontaneous pneumothorax. After surgery, two intercostal drainage tubes were needed due to a prolonged air leak; he was discharged after 15 postoperative days with a total hospital stay of 23 days. This case highlights the potential role of heavy marijuana and tobacco use in the development of recurrent spontaneous pneumothorax along with the increased risk of adverse outcomes and longer hospital stays in patients who smoke both substances. Further research is needed to better understand the underlying mechanisms and establish guidelines for the management of individuals with recurrent spontaneous pneumothorax who abuse marijuana.

## Conclusions

We aim to contribute to the existing evidence on the effects of combined marijuana and tobacco smoking on bullous lung disease and pneumothorax with our case report. Considering that the history of marijuana smoking, along with tobacco use, could lead to different outcomes in patients with primary pneumothorax, we strongly recommend screening all patients for marijuana use, especially those presenting apparent lung disease. A study has shown that asking a single question such as "Do you smoke marijuana?" or "Do you now, or have you ever smoked or used marijuana?" is the most effective screening tool for patients. Furthermore, it is crucial to educate and counsel patients about the harmful effects of smoking marijuana and underscore the dangers associated with combining it with tobacco.

## References

[1] Compton WM, Han B, Jones CM, Blanco C (2019). Cannabis use disorders among adults in the United States during a time of increasing use of cannabis. Drug Alcohol Depend.

[2] Dills AK, Goffard S, Miron J, Partin E (2021). The Effect of State Marijuana Legalizations: 2021 Update. Policy Analysis No. 908.

[3] Connor JP, Stjepanović D, Le Foll B, Hoch E, Budney AJ, Hall WD (2021). Cannabis use and cannabis use disorder. Nat Rev Dis Primers.

[4] Tashkin DP (2018). Marijuana and lung disease. Chest.

[5] Sahn SA, Heffner JE (2000). Spontaneous pneumothorax. N Engl J Med.

[6] Barton ED (1999). Tension pneumothorax. Curr Opin Pulm Med.

[7] Martinasek MP, McGrogan JB, Maysonet A (2016). A systematic review of the respiratory effects of inhalational marijuana. Respir Care.

[8] Stefani A, Aramini B, Baraldi C, Pellesi L, Della Casa G, Morandi U, Guerzoni S (2020). Secondary spontaneous pneumothorax and bullous lung disease in cannabis and tobacco smokers: a case-control study. PLoS One.

[9] Jain A, Ashiq A, Ahmed R, Rane RP, Hussain KM (2022). A case of pneumothorax secondary to marijuana use disorder. Cureus.

[10] Azzopardi M, Sladden D, Galea J (2021). Minimally invasive resection of a marijuana-associated giant bulla: a case report. Malta Med J.

[11] Manasrah N, Al Sbihi AF, Al Qasem S, Naik R, Hettiarachchi M (2021). Recurrent spontaneous pneumothorax associated with marijuana abuse: case report and literature review. Cureus.

[12] Aldington S, Williams M, Nowitz M (2007). Effects of cannabis on pulmonary structure, function and symptoms. Thorax.

[13] Tashkin D, Gliederer F, Rose J (1991). Tar, CO and THC delivery from the 1st and 2nd halves of a marijuana cigarette. Pharmacol Biochem Behavior.

[14] Hazouard E, Koninck JC, Attucci S, Fauchier-Rolland F, Brunereau L, Diot P (2001). Pneumorachis and pneumomediastinum caused by repeated Müller's maneuvers: complications of marijuana smoking. Ann Emerg Med.

[15] Luque MA 3rd, Cavallaro DL, Torres M, Emmanual P, Hillman JV (1987). Pneumomediastinum, pneumothorax, and subcutaneous emphysema after alternate cocaine inhalation and marijuana smoking. Pediatr Emerg Care.

[16] Rickert WS, Robinson JC, Rogers B (1982). A comparison of tar, carbon monoxide and pH levels in smoke from marihuana and tobacco cigarettes. Can J Public Health.

[17] Newton AS, Gokiert R, Mabood N (2011). Instruments to detect alcohol and other drug misuse in the emergency department: a systematic review. Pediatrics.

[18] Hedevang Olesen W, Katballe N, Sindby JE (2017). Cannabis increased the risk of primary spontaneous pneumothorax in tobacco smokers: a case-control study. Eur J Cardiothorac Surg.

[19] Ruppert AM, Perrin J, Khalil A (2018). Effect of cannabis and tobacco on emphysema in patients with spontaneous pneumothorax. Diagn Interv Imaging.

